# Dynamic chromatin accessibility deploys heterotypic *cis*/*trans*-acting factors driving stomatal cell-fate commitment

**DOI:** 10.1038/s41477-022-01304-w

**Published:** 2022-12-15

**Authors:** Eun-Deok Kim, Michael W. Dorrity, Bridget A. Fitzgerald, Hyemin Seo, Krishna Mohan Sepuru, Christine Queitsch, Nobutaka Mitsuda, Soon-Ki Han, Keiko U. Torii

**Affiliations:** 1 Howard Hughes Medical Institute, Department of Molecular Biosciences, The University of Texas at Austin, Austin, TX USA; 2 Department of Genome Sciences, University of Washington, Seattle, WA USA; 3 Bioproduction Research Institute, National Institute of Advanced Industrial Science and Technology (AIST), Tsukuba, Ibaraki, Japan; 4 Institute of Transformative Biomolecules (WPI-ITbM), Nagoya University, Nagoya, Aichi Japan; 5 Present Address: Department of New Biology, DGIST, Daegu, Republic of Korea

**Keywords:** Cell fate, Plant genetics

## Abstract

Chromatin architecture and transcription factor (TF) binding underpin cell-fate specification during development, but their mutual regulatory relationships remain unclear. Here we report an atlas of dynamic chromatin landscapes during stomatal cell-lineage progression, in which sequential cell-state transitions are governed by lineage-specific bHLH TFs. Major reprogramming of chromatin accessibility occurs at the proliferation-to-differentiation transition. We discover novel co-*cis* regulatory elements (CREs) signifying the early precursor stage, BBR/BPC (GAGA) and bHLH (E-box) motifs, where master-regulatory bHLH TFs, SPEECHLESS and MUTE, consecutively bind to initiate and terminate the proliferative state, respectively. BPC TFs complex with MUTE to repress *SPEECHLESS* expression through a local deposition of repressive histone marks. We elucidate the mechanism by which cell-state-specific heterotypic TF complexes facilitate cell-fate commitment by recruiting chromatin modifiers via key co-CREs.

## Abstract (web summary)

Chromatin accessibility dynamics profiling throughout the development of the stomatal lineage uncovers a crucial role for heterotypic *cis*- and *trans*-acting factors. These drive cell-fate commitment during specialized cell-type differentiation.

## Main

Differentiation of specialized cell types in multicellular organisms requires cell-state-specific, dynamic gene expression programmes governed by *cis*-acting regulatory DNA elements (CREs) and *trans*-acting factors (for example, transcription factors (TFs))^1,2^. Genome-wide identification of accessible chromatin through technologies such as the assay for transposase-accessible chromatin with high-throughput sequencing (ATAC-seq)^3,4^ have unravelled combinations of CREs that may signify specific developmental states^5–8^. In contrast to mammalian models, previous studies in plants have failed to demonstrate a strong correlation between chromatin openness and gene expression, leaving gaps in our understanding of the role of chromatin accessibility during cell-type differentiation^9,10^. Although studies have reported differentially accessible CREs in plants and animals^10–19^, how these motifs and TFs mutually influence gene expression to drive cell-fate decisions remains an important question.

Development of stomata—microscopic valves on the plant aerial epidermis for efficient gas-exchange and water control—has become an accessible model to understand de novo initiation and differentiation of lineage-specific cell types in plants^20,21^. A family of bHLH TFs: SPEECHLESS (SPCH), MUTE and FAMA, sequentially drive the initiation/proliferation, commitment and terminal differentiation of stomatal-lineage cells, respectively, by forming heterodimers with a partner bHLH, SCREAM (SCRM)^22–25^. Previous studies have provided repertoires of their direct target genes as well as transcriptomes in specific stomatal-lineage cell states^26–31^. However, it remains unknown how epigenomic state and stomatal TFs mutually interact to achieve cell-fate commitment during stomatal cell-lineage transitions.

By profiling dynamic chromatin accessibility and analysing genome-wide binding of stomatal-lineage regulators, we decipher the inventories of combinatorial *cis*/*trans* regulatory code of stomatal cell-lineage progression. Our multi-omics analysis identified the BBR/BPC (GAGA repeat) and bHLH (E-box) motifs as Co-CREs within the early stomatal lineage. Genetic, biochemical and biophysical analyses further unravelled the preferential association of BPC1/2 TFs with MUTE over SPCH, and that heterotypic BPC1/2-MUTE bHLH complex facilitates a local deposition of repressive chromatin marks. We propose the mechanism by which a cell-state-specific heterotypic TF complex achieves cell-fate commitment by taking over the shared accessible chromatin sites from a predecessor sister TF that maintains a proliferative state.

## Results

### Chromatin accessibility dynamics during stomatal development

**Fig. 1:**
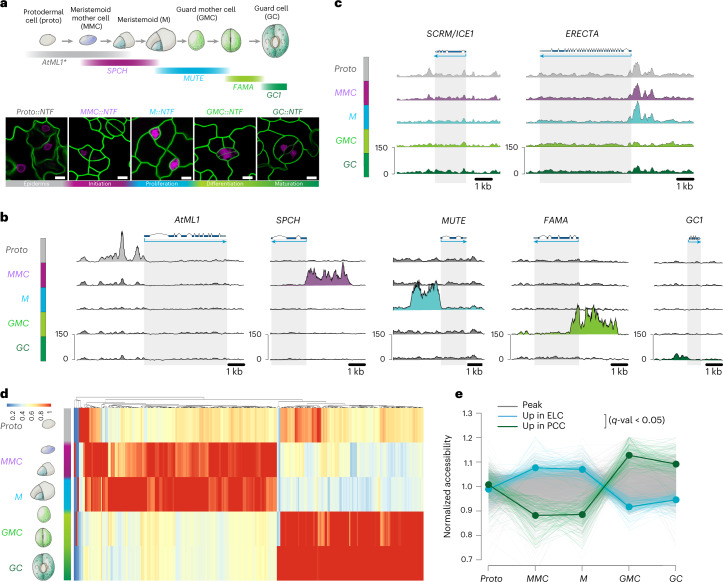
Dynamics of accessible chromatin landscape during stomatal cell-state progression. **a**, Schematic of stomatal development (top) with representative confocal images (bottom) of stomatal lineage cell-type-specific markers of each INTACT line (RFP) in LTi6b (GFP plasma membrane marker) background. These INTACT lines are used for monitoring the genome-wide chromatin accessibility by ATAC-seq. Each colour bar represents the duration of expression of each gene: *AtML1* (grey), *SPCH* (purple), *MUTE* (cyan), *FAMA* (light green), *GC* (green). *Note that *AtML1* is expressed predominantly in the protodermal cells, and its modest expression persists in the stomatal-lineage cells. Scale bar, 10 μm. The experiment was repeated independently three times with similar results. **b**, Genome browser view of differential chromatin accessibility sites showing the specificity of INTACT nuclei preparations. **c**, Genome browser view of static (*SCRM/ICE1*) and dynamic (*ERECTA*) chromatin accessibility sites of the endogenous locus encoding known regulators of stomatal development. **d**, Heat map showing differentially accessible sites during cell-state transition in stomatal development. For each THS, accessibility values are shown relative to the maximum value across stomatal developmental stage.**e**, A line plot trajectory of normalized chromatin accessibility shows two distinct clusters during stomatal development. ELC, early lineage specific cluster (cyan); PCC, post commitment cluster (green).

**Extended Data Fig. 1:**
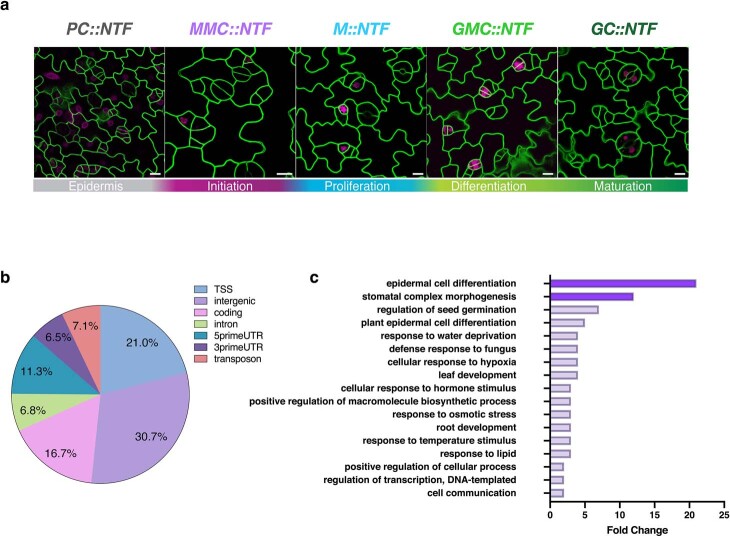
INTACT ATAC-seq. (**a**) Representative confocal images of stomatal lineage cell-type-specific markers of each INTACT line (RFP) in LTi6b (GFP plasma membrane marker) background. Shown are zoom-out view of Fig. 1a. Scale bars, 10 μm. The experiment was repeated independently three times with similar results. (**b**) Genomic distribution of THSs from stomatal lineage-specific ATAC-seq. THSs disproportionately located in intergenic, TSS, and 5′UTR elements. TSS, transcription start site. (**c**) Gene ontology analysis of dynamic THSs. Top 2 enriched categories were highlighted in purple.

To investigate the global chromatin landscape and its dynamics during stomatal-lineage transitions, we employed the stomatal cell-state-specific INTACT (Isolation of Nuclei TAgged in specific Cell Types) using our modified vector followed by ATAC-seq^3,4^ (Methods, Fig. 1 and Supplementary Table 1). Nuclei were captured from transient and terminal cells within the stomatal lineage: protoderm (Proto) (*AtML1*), meristemoid mother cells (MMCs) and early meristemoids (Ms) (*SPCH*), late Ms transitioning into guard mother cells (GMCs) (*MUTE*), GMCs transitioning into guard cells (GCs) (*FAMA*) and terminally differentiated mature GCs (*GC1*). Their correct stomatal-lineage-specific expression was confirmed by visualizing the INTACT marker red fluorescent protein (RFP) (Fig. 1 and Extended Data Fig. 1).

**Extended Data Fig. 2:**
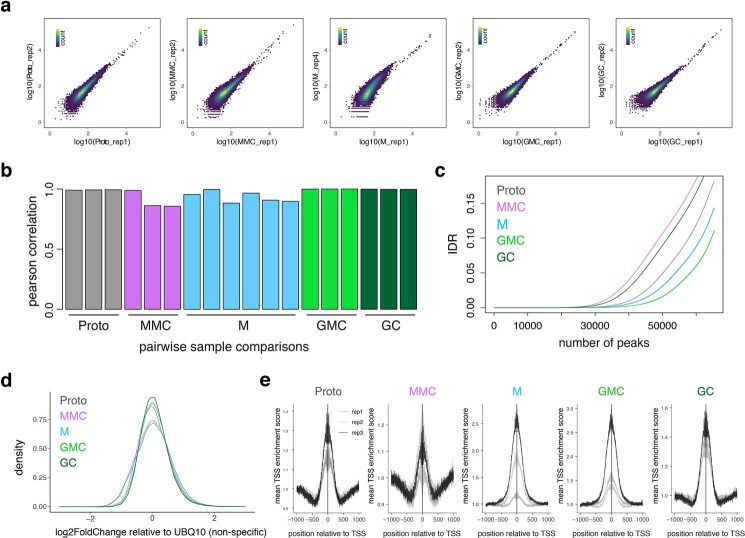
Quality evaluation and demonstration of reproducibility of INTACT-ATAC-seq data. (**a**) Scatterplots showing correlations of accessibility values (cutcounts per peak) between replicates in each sample. Density of overlapping points are shown as a color gradient. (**b**) Barplots showing Pearson correlation coefficient values for each pair of replicates in each sample (note that MUTE has four replicates, and therefore six pairwise correlations). (**c**) Line plot showing estimated irreproducible discovery rate (IDR) values for each sample as a function of peak ranking. (**d**) Density histogram showing magnitudes of accessibility differences from each sample relative to a non-specific, ‘bulk’ control sample. (**e**) TSS enrichment plots for each INTACT ATAC-seq sample showing, across all genes, the frequency of cutcounts at transcription start sites (shown as position 0), relative to flanking regions (1000 base pairs up or downstream). All samples show an expected peak at the TSS, with reduced signal in flanking regions. Replicates are shown in shades of grey for each sample.

From five cell-state-specific INTACT ATAC-seq libraries with 3-4 biological replicates for each, we identified 65,352 transposase hypersensitive sites (THSs), which are sites of open chromatin in stomatal-lineage trajectory cells. These THSs exhibit high reproducibility (average Pearson correlation *r* = 0.96 between biological replicates within each INTACT cell state) and an enrichment of transposition events at the transcription start sites (TSS) (Extended Data Figs. 1 and 2). They are commonly located in intergenic regions (30.7%) and ~400 bp upstream of TSS (21%) (Extended Data Fig. 1b). Such distribution of THSs is consistent with observations in *Drosophila*^32^ and in whole plant seedlings^10,11^, in which developmental enhancers exhibiting strong cell-type specificity reside near intergenic regions.

**Extended Data Fig. 3:**
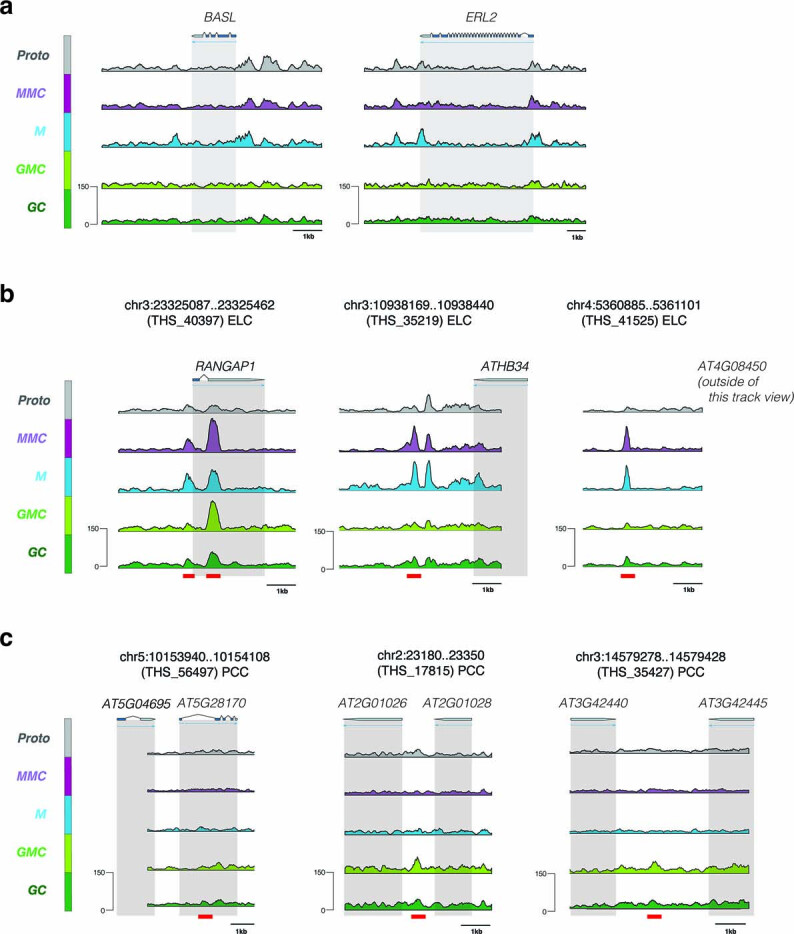
Genome browser view of differential chromatin accessibility sites. (**a**) Examples of THSs of early stomatal-lineage expressed genes, *BASL* (left) and *ERL2* (right). Location of genes are shaded in grey. The direction of transcription is indicated as a cyan arrow. Track height (y-axis) is indicated at the bottom track. All tracks are on the same scale. (**b**) Representative examples of THSs in the ELC. Red bars indicate the location of differentially accessible THSs highly open in ELC. (**c**) Representative examples of THSs in the PCC. Red bars indicate the location of differentially accessible THSs highly open in PCC.

Although most THSs are static, we identified 455 dynamic THSs that are differentially accessible within the stomatal lineages (*P* < 0.0005) (Methods, Extended Data Fig. 1 and Supplementary Tables 2 and3). Notably, strong yet highly dynamic peaks are found in the promoter region of stomatal-cell-state-specific genes utilized for INTACT (Fig. 1b). Importantly, we detected open chromatin peaks in the endogenous loci regulating stomatal development. For example, within the *SCRM* locus, which is known to be expressed throughout stomatal cell lineages^25^, static THSs are detected throughout all stages (Fig. 1c). In contrast, it is known that the receptor kinase gene *ERECTA* is highly and uniformly expressed in the protoderm, and while its expression persists in MMCs and Ms, it declines in GMCs and GCs^33,34^. Consistently, dynamic chromatin openings are detected within the promoter region of *ERECTA* in Proto, MMC and M, but they are diminished in GMC and GC (Fig. 1c). Likewise, other early stomatal-lineage genes exhibit prominent THSs in the early lineages (Extended Data Fig. 3a). These results suggest that temporal dynamics of chromatin accessibility and regulatory elements in these THSs may facilitate stomatal lineage transition. Indeed, overrepresented gene ontology (GO) categories of genes residing nearby dynamic THSs include epidermal cell differentiation (*P* = 0.000585), followed by stomatal complex morphogenesis (*P* = 0.000476; Extended Data Fig. 1).

**Extended Data Fig. 4:**
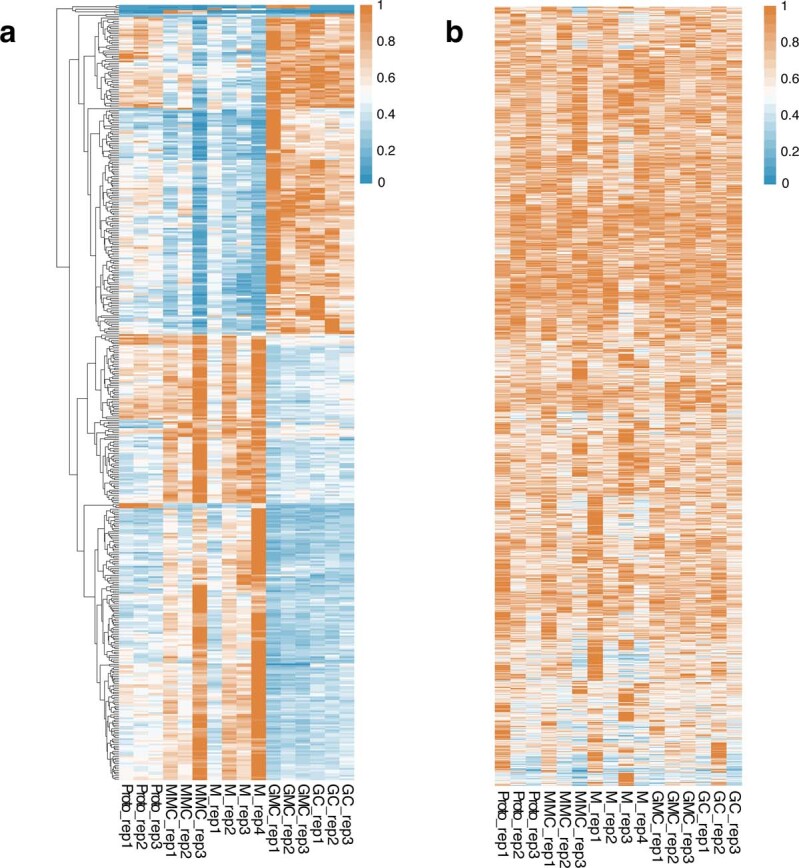
Variation in accessibility across replicates in dynamic and static THS. (**a**) Heatmap for THS identified as dynamic, as in Fig. 1d, with samples (split by replicate) shown on the x-axis. Values are normalized to the row maximum to show stages with greatest accessibility (orange). (**b**) Heatmap as in (a), but peaks are instead a sampling of non-significant peaks (p > 0.99) that show little variation across stages of stomatal development.

Hierarchical clustering analysis of the dynamic THSs revealed a profound change in global chromatin accessibility at the transition from Ms to GMCs (Fig. 1d). In contrast to the dynamic THSs, the static THSs did not exhibit any clear trends of clustering (Extended Data Fig. 4). This resulted in two major THS clusters: the early-lineage cluster (ELC) and the post-commitment cluster (PCC), which are accessible in the MMC/M stage and the GMC/GC stage, respectively (Fig. 1d,e; see Extended Data Fig. 3b,c for representative THSs in ELC and PCC, respectively). We also note here that the PCC is less dynamic than the ELC. Thus, the chromatin landscape may reduce complexity to prevent aberrant dedifferentiation of the terminal cell type, GCs^35^. In addition, we noted a small group of THSs in the protoderm. Since the number of protodermal THSs was too small for further analysis, we decided to focus on the two major clusters, PCC and ELC (Supplementary Table 3). Taken together, our INTACT ATAC-seq profiling elucidates the dynamic landscape of chromatin accessibility during successive cell-state transitions within the stomatal lineage and identifies sites of major epigenomic reprogramming at the commitment stage.

### A novel pair of *cis*-regulatory codes in early lineage

**Fig. 2:**
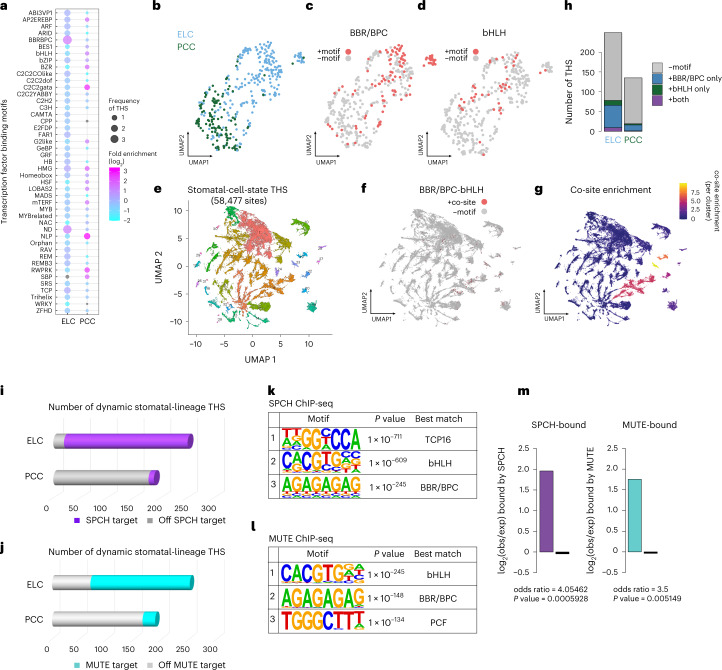
Identification of novel co-CREs in early stomatal precursor state through orthogonal INTACT ATAC-seq and ChIP-seq analyses. **a**, Enriched TF motifs identified from two distinct clusters in the stomatal lineage. Colour in dot blot graph denotes log_2_ ratio of changes in motif family frequency between clusters, and the size of the dot denotes relative frequency of each motif family in ELC and PCC clusters. **b**, UMAP dimensionality reduction plot of trajectory-specific THS sequences. An individual THS is represented as one data point, and data points are grouped by similar TF motif content. THSs that showed ELC-specific accessibility in INTACT ATAC-seq are coloured cyan, while those with PCC-specific accessibility are coloured green. **c**, UMAP plot as in **b**, showing individual THS data points containing a motif from the BBR/BPC family in red. THSs without a BBR/BPC motif are shown in grey. **d**, UMAP plot as in **a**, showing individual THS points containing a motif from the bHLH family in red. THSs without a bHLH motif are shown in grey. **e**, UMAP dimensionality reduction plot of all THSs detected in the INTACT ATAC-seq samples. Data points represent individual THSs and are grouped according to similarities in transcription factor motif content. Colours represent clusters of THS with similar motif content. **f**, UMAP plot as in **e**, showing individual THSs that contain motifs for both BBP/BPC family and bHLH family transcription factors. **g**, UMAP plot as in **e**, showing the enrichment of THSs with both BBR/BPC family and bHLH family motifs across clusters. Clusters 3, 19, 22 and 23 showed enrichments of THSs with motifs for both families. **h**, Barplot showing the total number of THSs in either the ELC- or PCC-specific set. **i**,**j**, Barplot showing the total number of dynamic THSs in either the ELC- or PCC-specific THSs targeted/not targeted by SPCH (**i**) or MUTE (**j**). The list of THSs is provided in Supplementary Table 6. **k**,**l**, De novo motif enrichment was performed with HOMER^79^ in SPCH ChIP-seq peaks (**k**) or MUTE ChIP-seq peaks (**l**). The top 3 motif results are shown with *P* values. **m**, log_2_ transformation of observed/expected number of dynamic THSs in each cluster bound by either SPCH (purple) or MUTE (cyan) (Fisher’s Exact test). ChIP-seq data adapted from refs. ^28,47^. In **k** and **l**, hypergeometric test was performed using HOMER (http://homer.ucsd.edu/homer/motif/index.html).

The drastic rearrangements of the chromatin landscape, as evident in the two major clusters of stomatal-lineage THSs, ELC and PCC (Fig. 1d,e), open the opportunity to identify *cis*-regulatory elements (CREs) represented in each state. We first performed motif analysis using FIMO to search for significant (adjusted *P* < 1 × 10^−5^) matches for each motif within accessible sites^36^ (Methods). Of 65,351 stomatal-lineage accessible sites, 58,477 (89.5%) contain a significant motif match (Fig. 2a,e). Within the ELC, the most significantly enriched motif is BBR/BPC (BARLEY B RECOMBINANT/BASIC PENTACYSTEINE), also known as the GAGA repeat comprising tandem repeats of GA (Fig. 2a). The second most enriched motif, ND, also includes GAGA repeat sequences as a consensus^37^. In the PCC, C2C2-GATA, RWP-RK and NLP motifs are enriched, implying that the major rewiring of gene regulatory networks may occur at commitment (Fig. 2a). Consistent with the FAMA-driven guard-cell maturation process, GATA-binding TFs are known to promote chloroplast development and plant greening^38^.

The enrichment of BBR/BPC motif CREs in ELC THSs captured our attention. It is established that a plant-specific BBR/BPC family of transcription factors bind to the BBR/BPC motif^39–44^. To test whether the specific CREs we isolated are bound by BBR/BPC, we performed an unbiased yeast one-hybrid (Y1H) screen^45^ to isolate a suite of TFs binding to the highly ranked THSs in the ELC. Indeed, Class I BPC proteins (BPC1, 2, 3) were isolated as strong interactors (Extended Data Fig. 6).

**Extended Data Fig. 5:**
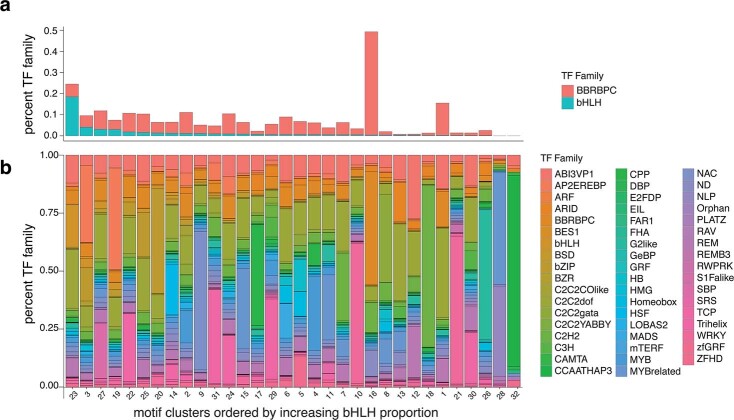
Transcription factor motif enrichment in THS clusters. (**a**) Barchart showing fraction of BPC and bHLH TF families in each THS cluster, sorted by bHLH fraction. (**b**) Stacked barchart showing fraction of all TF families represented in each THS cluster, with ordering as in (a). Each TF family is shown as a different colour.

Combinatorial *cis*-regulatory motifs may guide the specificity of transcription factor binding during cell-state transition. To explore this possibility, we applied the uniform manifold approximation and projection (UMAP) dimensionality reduction algorithm^46^ to a count matrix for all motifs observed in all THSs, allowing THSs with similar motif contents to be grouped in two dimensions (Fig. 2b–g) (Methods). Each data point represents an individual THS, and data points are clustered according to similarities in transcription factor motif content. We used this approach first to examine THSs belonging to ELC and PCC (Fig. 2b–d). Surprisingly, the UMAP revealed that BBR/BPC and the bHLH (E-box) motifs are significantly co-enriched compared with the background, and co-occurrence of these two motifs is notable in ELC THSs (Fig. 2h). We also examined the co-occurrence of BBR/BPC and bHLH motifs in the UMAP generated from all THSs identified in stomatal lineages and found that multiple clusters of THSs were enriched for this pair of motifs, suggesting that these TFs may indeed act together at specific CREs (Fig. 2e–g; for all TF motifs enriched in each THS cluster, see Extended Data Fig. 5). This hints at a possible co-action of two disparate CREs (BBR/BPC and bHLH motifs) as well as the Class I BPC *trans*-acting proteins during the early stomatal cell lineage. Such co-action may facilitate the transient state, sequentially regulated by the two bHLHs, SPCH and MUTE, which specify stomatal precursor state.

### Early-lineage THS cluster controlled by two sister bHLHs

The early stomatal cell lineage is initiated by SPCH and terminated by its paralogue, MUTE—two sister stomatal-lineage-specific bHLHs expressed in MMCs/early meristemoids and late meristemoids/GMCs, respectively^23,24^. Nonetheless, within the ELC, the chromatin landscape is largely similar, indicating that these two bHLHs probably share a set of binding sites. As an orthogonal approach to cistrome discovery via INTACT ATAC-seq, we leveraged chromatin Immunoprecipitation sequencing (ChIP-seq) of SPCH^28^ and MUTE^47^ to directly profile transcription factor occupancy in relationship with the dynamic stomatal-lineage THSs. Nearly 86% and >70% of the ELC THSs are targeted by SPCH and MUTE, respectively, whereas only 4.78% and 12% of the later PCC THSs are targeted by SPCH and MUTE, respectively (Fig. 2i,j). Thus, the vast majority of dynamic open chromatin regions in the early stomatal lineage cells are occupied by both SPCH and MUTE.

**Extended Data Fig. 6:**
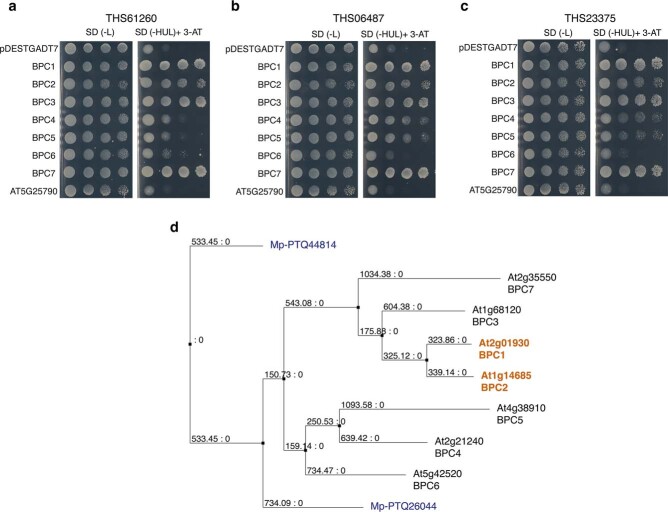
BPC TFs bind to the ELC-specific THS. (**a-c**) Yeast one hybrid analysis showing class I BPCs and BPC7 bind to ELC specific THSs, THS61260 (**a**), THS06487 (**b**), and THS23375 (**c**). AT5g25790 and pDESTGADT7 are negative control in this study. THS61260 is located within the promoter of *SPCH*. (**d**) Phylogenetic tree analysis of Arabidopsis BPCs. Full amino acid sequences were subjected to Neighbor Jointing analysis with Percent Accepted Mutation (PAM) 250 matrices. *Marchantia polymorpha* BPC proteins were used as an outgroup.

**Extended Data Fig. 7:**
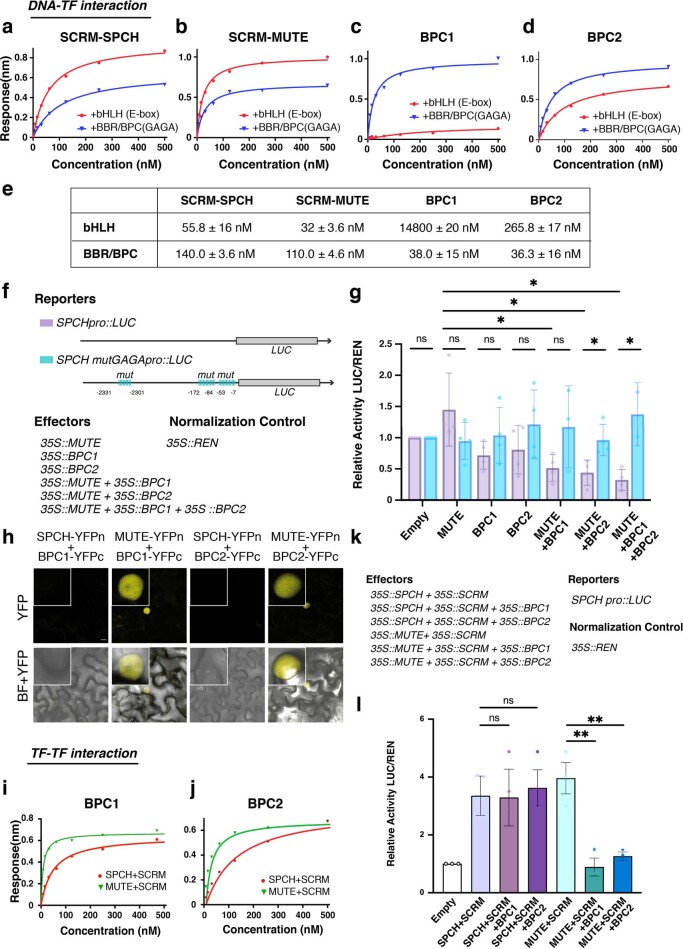
BPCs repress *SPCH* expression together with MUTE via BBR/BPC motif. (**a-d**) Quantitative analysis of bHLH (E-box) and BBR/BPC (GAGA) motif DNA-TF interactions. Interaction between SCRM-SPCH (a), SCRM-MUTE (**b**), BPC1 (**c**), and BPC2 (**d**) with bHLH (E-box) (red) and BBR/BPC (GAGA) (blue) motif using BLI. Data are mean ± s.d., (n = 2) (**e**) Table of *Kd* value calculated from the BLI. (**f**) Schematics of dual-luciferase assays performed in this study. (**g**) Dual-luciferase assays of *SPCH* promoter and *SPCH* promoter with mutation in BBR/BPC motif with individual or pairwise combinations of effector MUTE, BPC1, and BPC2. Relative luciferase activity (LUC/REN) was normalized against empty vector control. Three biological replicates were performed, each with 3 technical replicates. Error bars = s.e.m. (n = 3). Student’s t-test was performed; *, p < 0.05; ns, not significant. (**h**) BiFC assay showing BPC1 and BPC2 physical interaction with MUTE, but not with SPCH. *N. benthamian* leaves were agroinfiltrated using pairwise combinations of each construct as indicated. YFP, confocal imaging of YFP signal; BF, bright field; inset, magnified image of a representative nucleus. Scale bar, 10 μm. The experiment was repeated independently three times with similar results. (**i**, **j**) Quantitative analysis of BPC1 (i) and BPC2 (j) with MUTE-SCRM (green) compared to SPCH-SCRM (red) interaction by BLI. Shown are i*n vitro* binding response curves for purified BPC1, BPC2, MUTE-SCRM, and SPCH-SCRM protein at seven different concentrations (500, 250, 125, 62.5, 31.25, 15.625, 7.8125 nM). Data are mean ± s.d., (n = 2). (**k**) Schematics of dual-luciferase assays performed in this study. (**l**) Dual-luciferase assays of *SPCH* promoter with SCRM and individual or pairwise combinations of effectors: SPCH, MUTE, BPC1, and BPC2. Relative luciferase activity (LUC/REN) was normalized against empty vector control. Three biological replicates were performed, each with 3 technical replicates. Error bars = s.e.m (n = 3). Student’s t-test was performed; *, p < 0.05; **, p < 0.005; ns, not significant. See Supplementary Table 9 for all exact p values.

Next, to explore a possible connection of SPCH/MUTE binding motifs with the CREs identified in our analyses (Fig. 2), we performed de novo discovery of enriched motifs in the SPCH and MUTE binding peaks (Methods). As expected, the bHLH (E-box) motif was identified as among the top scoring motifs in MUTE and SPCH binding peaks (Fig. 2k,l). Strikingly, the BBR/BPC (GAGA repeat) motif, which is highly enriched in ELC THSs and co-occurs with the bHLH motifs (Fig. 2a,h), is the next highest enriched motif in both SPCH and MUTE peaks (Fig. 2k–m). SPCH and MUTE targets that possess BBR/BPC and bHLH motifs are overrepresented in ELC compared with PCC (Fig. 2m and Supplementary Tables 4–6). Unlike BPC proteins, however, neither SPCH nor MUTE protein binds to the BBR/BPC motif in our Y1H screen or in in vitro DNA-TF binding assays using biolayer interferometry (BLI) (Extended Data Figs. 6 and 7a–e). As predicted, SPCH and MUTE, when heterodimerized with SCRM, both bind tightly to the bHLH (E-box) motif (Extended Data Fig. 7a–e). The results imply that SPCH/MUTE binding to the BBR/BPC motif is indirect. Taken together, the two orthogonal approaches—discoveries of cell-state-specific CREs via INTACT ATAC-seq and TF binding sites via ChIP-seq analyses—highlight Class I BPCs as possible *trans*-acting regulators of MMC- and/or MC-mediated cell-state transition.

### BPC1/2 suppress *SPCH* and early stomatal-lineage divisions

**Fig. 3:**
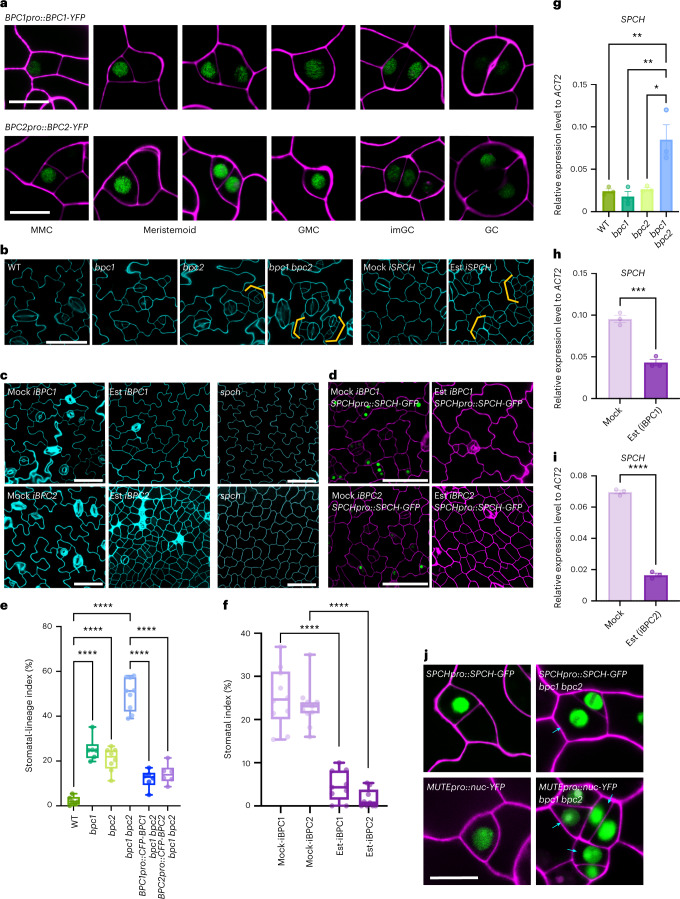
New *trans*-acting factors, *BPC1* and *BPC2*, suppress stomatal-lineage entry division and *SPCH* expression. **a**, Confocal images showing the expression pattern of *BPC1* and *BPC2* translational reporters in stomatal-lineage cells. YFP signal is depicted in green and cell walls in magenta. Scale bar, 10 μm. **b**, Epidermal phenotype of abaxial cotyledon in 8-day-old WT (wild-type), *bpc1*, *bpc2* and *bpc1 bpc2* double mutants. Scale bar, 50 μm. Orange brackets, excessive cell division. **c**, Epidermal phenotype of abaxial cotyledon in 5 days after germination (dag) inducible line of BPC1 and BPC2 compared to Mock. The phenotypes in the inducible line of BPC1 and BPC2 are reminiscent of those of the 3 dag and 1 dag *spch* mutant epidermis, respectively. Scale bar, 50 μm. **d**, iBPC1 and iBPC2 trigger repression of *SPCHpro::SPCH-GFP*. GFP signal is depicted in green and cell walls in magenta. Scale bar, 50 μm. **e**, Stomatal lineage index (SLI) of abaxial cotyledon epidermis in 8 dag WT, *bpc* mutants and *bpc* mutants genetically complemented with respective *BPC* genes driven by its promoter (*n* = 6). SI data are provided in Extended Data Fig. 8b. **f**, SI of abaxial cotyledon epidermis of control mock and inducible line of *BPC1* and *BPC2* (*n* = 6). For the boxplots in **e** and **f**, the centre line indicates the median value, and the whiskers represent 1.5× the interquartile range from the lower and upper quartiles. For **e**–**g**, one-way ANOVA followed by Tukey’s HSD test was performed: *P < 0.05, **P < 0.005, ****P < 0.00005. **g**–**i**, Relative expression analysis for early stomatal precursor marker genes in WT, *bpc* mutants and BPC inducible line. Loss of *BPC1* and *BPC2* derepresses the expression of *SPCH* (**g**) as well as early stomatal precursor marker genes (see Extended Data Fig. 9), while *SPCH* expression is repressed by iBPC1/2 (**h**,**i**). Expression was normalized against actin (*ACT2*). For **h** and **i**, two-tailed Student’s *t*-test was performed for pairwise comparisons: ***P < 0.0005, ****P < 0.00005. Error bars, s.e.m. (*n* = 3) **j**, Reporter marker genes, *SPCHpro::SPCH-GFP* and *MUTEpro::nucYFP*, in *bpc1 bpc2*. GFP and YFP signals are depicted in green and cell walls in magenta. Scale bar, 10 μm. Cyan arrowhead, ectopic expression. Experiments in **a**–**d** and **j** were repeated independently three times with similar results. See Supplementary Table 9 for all exact *P* values.

BPC proteins are expressed broadly and are known to regulate multiple developmental processes^40,48^. A previous single-cell transcriptome study of *Arabidopsis* noted that *BPCs* positively regulate stomatal development, probably via activating *SPCH*, *MUTE*, *FAMA* and *SCRM*^30^. However, we identified the BBR/BPC motif as a significantly overrepresented CRE in ELC, co-existing with a SPCH/MUTE binding site (Fig. 2). For this reason, we sought to decipher the exact functions and the mechanism of action of BPC1/2 during stomatal cell-state transition. First, we examined the expression patterns of BPC1 and BPC2 within the stomatal lineages. Both BPC1 and BPC2 fused with yellow fluorescent protein (YFP) driven by their respective promoters (*BPC1pro::BPC1-YFP* and *BPC2pro::BPC2-YFP*) showed strong signals in the nuclei of stomatal-lineage cells, with the highest expression from early meristemoid to GMC (Fig. 3a). We thus postulated that cell-state-specific, combinatorial interactions among *Trans*-acting factors (SPHC, MUTE and BPCs) could facilitate cell-fate transitions even under a similar chromatin configuration.

**Extended Data Fig. 8:**
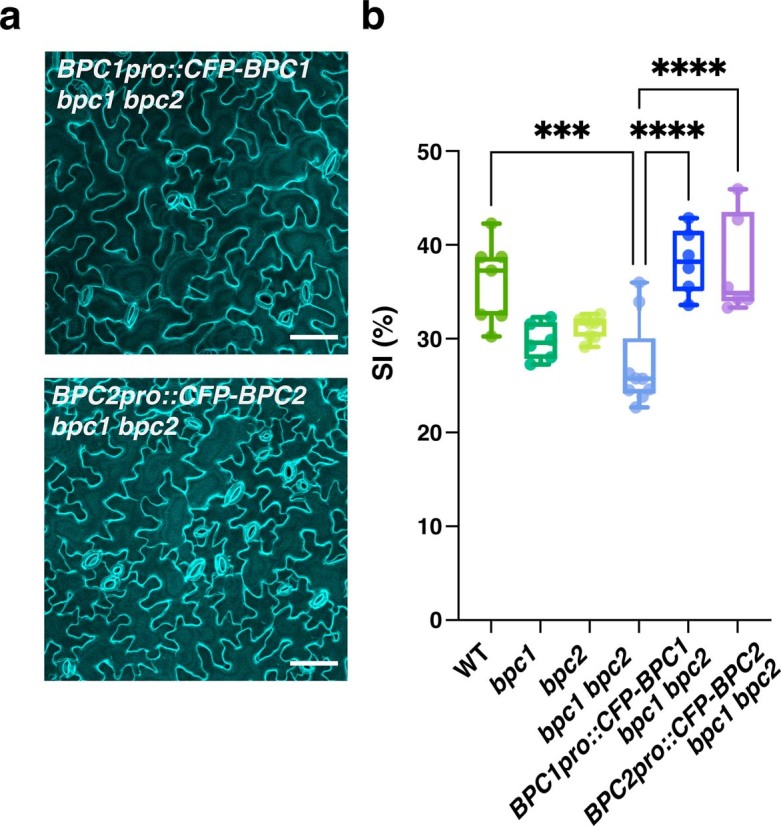
Characterization of *bpc1, bpc2, bpc1 bpc2* and complementation test. (**a**) Complementation analysis. Epidermal phenotype of abaxial cotyledon in 8day old *BPC1pro::CFP-BPC1* in *bpc1bpc2* and *BPC2pro::CFP-BPC2* in *bpc1 bpc2* double mutant; scale bar, 50 μm. The experiment was repeated independently three times with similar results. (**b**) Stomatal Index (SI) of abaxial cotyledon in WT, *bpc* mutants, and complimentary line. One-way ANOVA test was performed followed by Tukey’s HSD test was performed: ***, p < 0.0005; ****, p < 0.00005. The number of plants from each genotype is 6 (n = 6). The box plot boundaries reflect the interquartile range. The centre line indicates the median value, and the whiskers represent 1.5× the interquartile range from the lower and upper quartiles. See Supplementary Table 9 for all exact p values.

**Extended Data Fig. 9:**
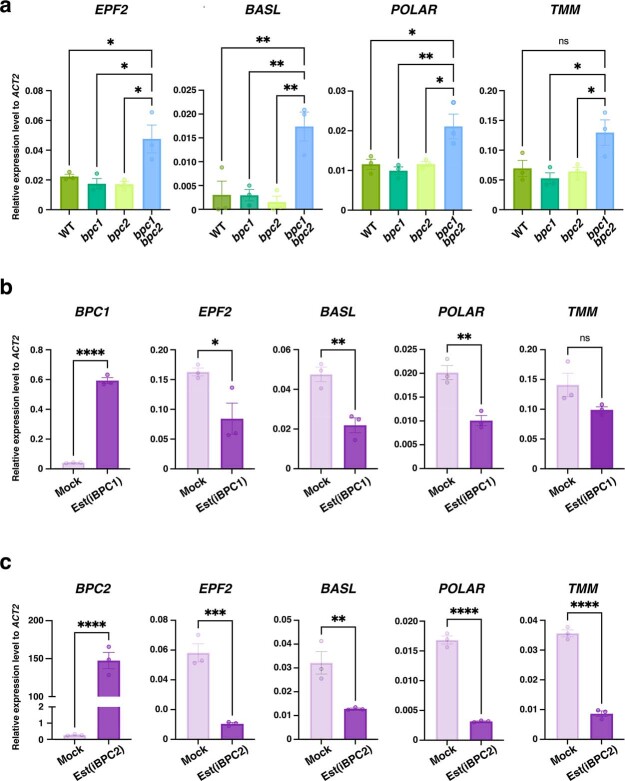
Relative expression analysis for early stomatal precursor marker genes in WT, *bpc* mutants, and BPC inducible overexpression lines. (**a**) Loss of *BPC1* and *BPC2* induce the expression early stomatal precursor marker genes, *EPF2, BASL, POLAR*, and *TMM*. Conversely, they are repressed by induced overexpression of iBPC1 (**b**) and iBPC2 (**c**). Relative expression represents qRT-PCR values normalized over that of *ACTIN2*. Student’s t-test was performed for pairwise comparisons: *, p < 0.05; **, p < 0.005; ***, p < 0.0005; ****, p < 0.00005; ns, not significant. Error bars, s.e.m. (n = 3) Three biological replicates were performed, each with 3 technical replicates. See Supplementary Table 9 for all exact p values.

Next, we characterized loss-of-function phenotypes of *BPC1* and *BPC2*. Both *bpc1* and *bpc2* showed modest increases in the number of early stomatal precursors, with statistical significance in the stomatal-lineage index (SLI = (number of meristemoid mother + meristemoid + SLGC)/number of all epidermal cells × 100) (Fig. 3b,e and Extended Data Fig. 8). The phenotype was further exaggerated in the *bpc1 bpc2* double mutant, suggesting their redundant activity (Fig. 3b,e and Extended Data Fig. 8). Excess early stomatal-lineage cells in *bpc1 bpc2* resembles the *SPCH* overexpression phenotype (Fig. 3b)^23,24,29^. Indeed, quantitative PCR with reverse transcription(RT-qPCR) analyses detected elevated transcript levels of *SPCH* and its direct targets (*EPF2, POLAR* and *BASL*) in the *bpc1 bpc2* mutants (Fig. 3g and Extended Data Fig. 9). In addition, asymmetric divisions of stomatal-lineage cells are dysregulated in *bpc1 bpc2*; the late meristemoid-fate marker *MUTEpro::nucYFP*^24,49^ is occasionally expressed in both daughter cells of asymmetric divisions (Fig. 3j). These inappropriate daughter cells eventually lose stomatal-lineage fate and fail to differentiate. The *bpc1 bpc2* epidermal phenotype was rescued by the introduction of either *BPC1* or *BPC2* driven by their respective promoter (Extended Data Fig. 9).

In contrast to the loss-of-function phenotypes, estradiol-inducible overexpression of *BPC1* and *BPC2* (*iBPC1* and *iBPC2*) led to an epidermis with seemingly uniform pavement cells with a significantly reduced stomatal index (SI = number of stomata/(number of stomatal + non-stomatal epidermal cells) × 100) (Fig. 3c,f). The *iBPC2* seedlings exhibited growth defects and failed to form lobed pavement cells (Fig. 3c,d). These phenotypes are reminiscent of the *spch* mutant^23,24^ (Fig. 3c). Indeed, both *SPCH* transcripts and SPCH-GFP reporter expression are severely diminished in the *spch* mutant-like *iBPC1* and *iBPC2* overexpression epidermis (Fig. 3d,h,i). Combined, our findings place BPC1 and BPC2 as *trans*-acting factors that suppress entry and proliferative divisions of stomatal-lineage cells via negative regulation of *SPCH* expression.

### Fate commitment by MUTE-BPC1/2 and repressive histone marks

Whereas SPCH and MUTE both promote stomatal differentiation, they act in an antagonistic manner on early stomatal precursor cells: SPCH initiates and promotes asymmetric divisions whereas MUTE terminates the asymmetric division and triggers differentiation, in part via displacing SPCH-binding sites^23,24,29^. To understand the mechanism by which BPC1/2 repress *SPCH* expression in the context of the stomatal cell-state transition, we sought to test our hypothesis that BPC1/2 TFs recruit MUTE to the ELC open chromatin sites via binding to the BBR/BPC (GAGA repeat) motif in a cell-state-specific manner.

**Fig. 4:**
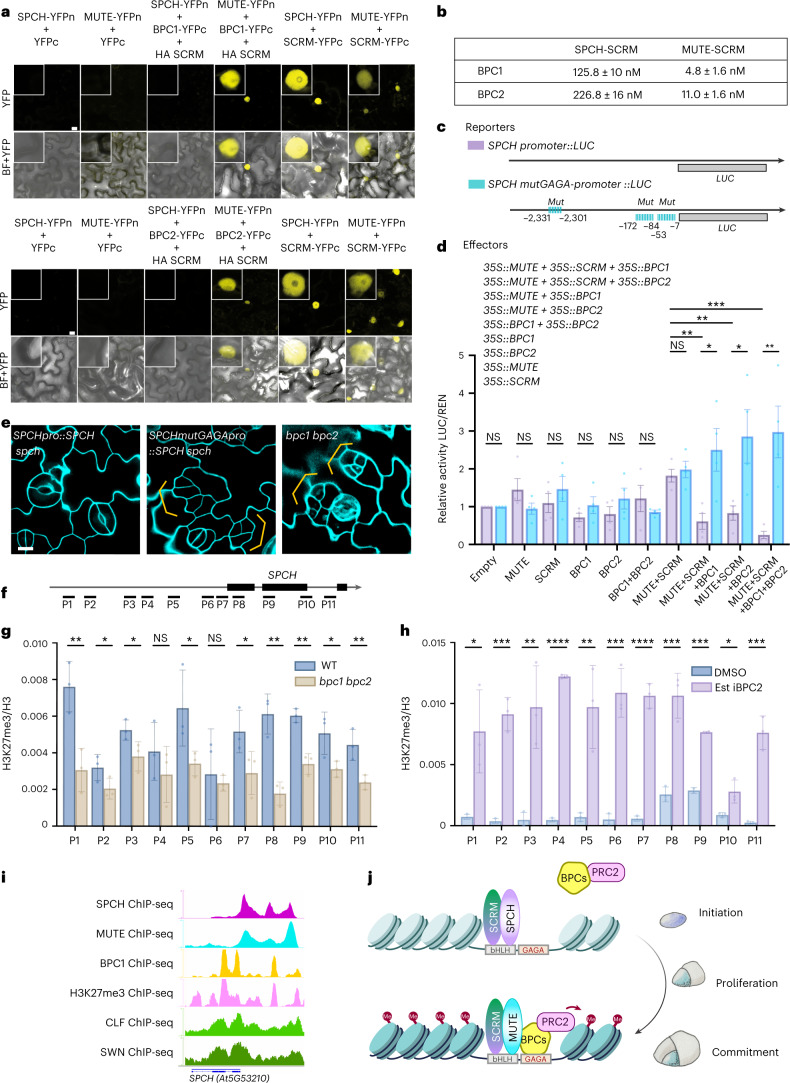
BPC1/2 physically interact with MUTE-SCRM to repress *SPCH* by recruiting H3K27me3. **a**, BiFC assay showing BPC1 and BPC2 physical interaction with MUTE-SCRM, but not with SPCH-SCRM. YFP, confocal imaging of YFP signal; BF, bright field. Inset: magnified image of a representative nucleus. Scale bar, 10 μm. The experiment was repeated independently three times. **b**, Table of *K*_d_ values calculated from BLI experiments that quantitatively analysed the interaction of BPC1 and BPC2 with SPCH-SCRM and MUTE-SCRM, individually. **c**, Schematics of dual-luciferase assays performed in this study. **d**, Dual-luciferase assays of *SPCH* promoter and *SPCH* promoter with mutation in BBR/BPC motif with individual or pairwise combinations of effector MUTE, BPC1, BPC2, BPC1 + BPC2 and SCRM. Relative luciferase activity (LUC/REN) was normalized against empty vector control. Error bars, s.e.m. (*n* = 3). **e**, Confocal microscopy images of *spch* mutant complemented by the introduction of *SPCHpro::SPCH* (left) and hyperactive entry divisions (orange brackets) by *SPCHmutGAGApro::SPCH* (middle), which resembles *bpc1 bpc2* (right) mutant epidermis. Scale bar, 10 μm. The experiment was repeated independently three times with similar results. **f**, Primer location of SPCH used for ChIP-qPCR analysis (Supplementary Table 8). **g**, ChIP assay of H3K27me3 accumulation level (normalized to H3 to control for nucleosome density) at the *SPCH* locus in the aerial part of 8-day-old WT and *bpc1 bpc2* double mutant. **h**, ChIP assay of H3K27me3 accumulation level (normalized to H3 to control for nucleosome density) at the *SPCH* locus upon estradiol induction compared to Mock. For **g** and **h**, Student’s *t*-test was performed for pairwise comparisons: *P < 0.05, **P < 0.005, ***P < 0.0005, ****P < 0.00005; NS, not significant. Error bars, s.e.m. (*n* = 3). **i**, Genome browser view of ChIP-seq profile of SPCH, MUTE, BPC1, CLF, SWN and H3K27me3 binding to the *SPCH* locus. ChIP-seq data adapted from ref. ^52,80^. **j**, Schematic model. The SPCH-SCRM module binds to the E-box in the *SPCH* promoter to initiate asymmetric divisions. At proliferation stage, the MUTE-SCRM module binds to the BBR/BPC (GAGA) motif mediated by BPC1/2 to terminate asymmetric division. Therefore, BPCs can recruit PRC2 complex to *SPCH* loci to alter chromatin structure through repressive histone mark deposition (Me, H3K27me3). Different combinations of lineage-determining transcription factors might specify the genomic sites that are responsible for cell-fate transition. See Supplementary Table 9 for all exact *P* values.

We first examined whether BPC1/2 bind with MUTE in planta using bimolecular fluorescent complementation (BiFC) assays in *Nicotiana benthamiana* (Fig. 4a and Extended Data Fig. 7h). Co-expression of a full-length BPC1 or BPC2 fused to the half YFP (YFPc) and MUTE to the complementary half YFP (YFPn) yielded reconstitution of YFP signals in the nucleus, indicating that BPC1/2 interact with MUTE in the nucleus. By contrast, we detected no signals of SPCH and BPC1/2 combinations (Extended Data Fig. 7h), suggesting that SPCH does not interact with BPC1/2. SPCH and MUTE both function as bHLH heterodimers with SCRM^25,50^. Indeed, both SPCH-YFPn and MUTE-YFPn reconstituted strong YFP signals with SCRM-YFPc (Fig. 4a), thereby confirming that SPCH-YFPn proteins are expressed in *N. benthamiana*. We subsequently tested whether the presence of SCRM intensifies the interaction of MUTE (or SPCH) with BPC1/2. Strong YFP signals are reconstituted when non-fluorescent-protein-tagged SCRM is co-expressed with BPC1/2 and MUTE, but not SPCH (Fig. 4a). Thus, SPCH does not associate with BPC1/2 even in the presence of SCRM. Next, we performed in vitro quantitative binding kinetic assays of BPC1/2 with MUTE-SCRM heterodimers using BLI (Fig. 4b and Extended Data Fig. 7i,j). BPC1 and BPC2 associated very tightly with MUTE-SCRM (4.8 ± 1.6 nM and 11.0 ± 1.6 nM, respectively). In contrast, SPCH-SCRM heterodimers showed negligible interaction with BPC1/2 (Fig. 4b and Extended Data Fig. 7i,j). Combined, both our BiFC and BLI results suggest that BPC1/2 specifically function with the MUTE-SCRM module in the meristemoid stage exclusively.

To unravel the mechanism of *SPCH* repression via BPC1/2, we harnessed dual-luciferase assays in *N. benthaminana* using the reporter *Luciferase* fused with a native *SPCH* promoter, which possesses three BBR/BPC (GAGA repeat) and bHLH motifs (Fig. 4c and Extended Data Fig. 7f,g). Individual introduction of BPC1, BPC2, MUTE and SCRM alone, as well as co-introduction of BPC1 and BPC2 together alone had negligible effects on *SPCHpro::Luc* activity. As expected, *LUC* activity was elevated when MUTE and SCRM were co-expressed, indicating that MUTE-SCRM heterodimers function as transcriptional activators (Fig. 4d). Strikingly, co-expression of BPC1, BPC2 or BPC1 BPC2, together with MUTE and SCRM, led to strong and significant repression of *LUC* activity (Fig. 4d). However, when GAGA repeats were mutated (*SPCHmutGAGApro::LUC*), *LUC* activities became derepressed, or even became higher, in the presence of BPC1/2 and MUTE-SCRM (Fig. 4d and Extended Data Fig. 7f,g). The results suggest that MUTE-SCRM, which can act as transcriptional activators, repress gene expression via BBR/BPC (GAGA repeat) *cis*-element-mediated recruitment through BPC1 and BPC2. We further tested whether BPC1/2 repress the transcriptional self-activation of *SPCH* by SPCH-SCRM heterodimer (Extended Data Fig. 7k,l)^34^. Strikingly, co-expression of SPCH-SCRM with BPC1 as well as BPC2 had no effects on the reporter *SPCHpro::Luc* activity (Extended Data Fig. 7k,l). The results support the hypothesis that BPC1/2 must be recruited by MUTE to repress *SPCH* expression. Lastly, we tested whether the *SPCH* promoter with mutated GAGA motif confers excessive SPCH activities. Indeed, whereas control *SPCHpro::SPCH* rescued the *spch* mutant to the wild-type phenotype, *SPCHmutGAGApro::SPCH* in *spch* led to excessive stomatal-lineage entry divisions, phenocopying the *bpc1 bpc2* double mutant (Fig. 4e). This finding provides in vivo evidence for the repression of *SPCH* by BPC1/2 via the BBR/BPC (GAGA repeat) motif.

How does BPC1/2 repress *SPCH* expression in cooperation with MUTE-SCRM? A recent study showed that plant Class I BPC proteins and animal GAGA-binding proteins function similarly in the recruitment of the Polycomb Repressive Complex 2 (PRC2)^41,51^. The BPC that binds to the BBR/BPC motif physically interacts with and recruits PRC2, and this recruitment is required for in vivo PRC2-mediated gene silencing through Histone H3 Lys27 trimethylation (H3K27me3)^41,51,52^. Whereas it is well recognized that transcription factors jointly recruit PRC2 to the *cis* elements, known as Polycomb response elements (PREs)^52–54^, it remains unclear how cell-state-specific open chromatins, specialized transcription factors and repressive histone mark deposition together drive the switch from proliferation to differentiation.

To address the hypothesis that PRC2 contributes to the repression of *SPCH* expression via recruitment through BPC1/2, we first evaluated the level of H3K27me3 deposition at the *SPCH* locus in wild-type vs *bpc1 bpc2* seedlings by ChIP-qPCR assays (Fig. 4f,g). In the wild-type seedlings, H3K27m3 was deposited in both the promoter and gene body of *SPCH*. In contrast, the level of H3K27me3 was significantly reduced in *bpc1 bpc2* mutants (Fig. 4g). Next, we explored the level of H3K27me3 deposition at the *SPCH* locus upon induced *BPC2* overexpression, which confers stronger *spch*-like phenotype (Fig. 3c,d). Notably, the enrichment level of H3K27me3 was significantly increased in the promoter and gene body, especially around BBR/BPC motifs near TSS (Fig. 4h). Consistently, publicly available ChIP-seq data of BPC1 and H3K27me3^52^ showed nearly identical binding peak patterns within the *SPCH* locus at the proximity of SPCH and MUTE binding peaks (Fig. 4i). Furthermore, robust binding peaks of PRC2 components, CURLY LEAF (CLF) and SWINGER (SWN) are detected in the *SPCH* locus (Fig. 4i). Taken together, we conclude that BPC proteins recruit PRC2 to the *SPCH* locus via BBR/BPC motifs and consequently represses *SPCH* expression by catalysing the repression histone marks (H3K27me3) to drive stomatal cell-state transition from proliferation to differentiation, together with MUTE (Fig. 4j).

## Discussion

In this study, we have revealed chromatin accessibility dynamics and obtained the lexicon of *cis*-regulatory codes during stomatal cell-lineage progression. Integration of INTACT ATAC-seq and ChIP-seq of SPCH and MUTE highlighted BBR/BPC (GAGA repeat) and bHLH (E-box) motifs as early-lineage co-CREs (Figs. 1 and 2). By taking the multi-faceted approaches of molecular-genetic, biochemical and biophysical quantitative TF-TF and TF-DNA interaction assays, we demonstrate that a bHLH heterodimer, MUTE-SCRM, cooperates with BPC1/2 to achieve cell-fate commitment (Figs. 3 and 4).

Whereas chromatin accessibility and SPCH/MUTE binding sites are largely similar within the early stomatal lineage (Fig. 1d), our work suggests the occurrence of drastic changes in core TF complex behaviours. During proliferation, negligible association of BPCs with SPCH-SCRM probably permits SPCH to bind to the E-box and activate downstream gene expression for the asymmetric proliferating divisions. Upon commitment to differentiation, tight heterotypic interactions of BPCs with MUTE-SCRM probably usher this bHLH heterodimer to BBR/BPC motifs within desired genomic locations, as gated by chromatin accessibility, thereby facilitating a smooth transition of gene expression programmes into the GMC state (Fig. 4j).

Such heterotypic TF complex is also observed during cardiogenesis, hematopoiesis and myogenesis^9,55–59^. For instance, heterotypic TF interactions among the T-box TF TBX5, the homoeodomain TF NKX2-5 and the zinc finger TF GATA4 not only coordinate cardiac gene expression, differentiation and morphogenesis, but also mutually limit their potential from binding to developmentally irrelevant regulatory elements in a given context^9,55^. In another example, the heterotypic complex of myogenic bHLH proteins, Myo-D, and the myocyte enhancer MADS domain TF MEF2 also cooperatively regulate initiation of myogenesis^60–63^. Versatile heterotypic TF interactions with combinatorial CREs may serve as a paradigm for gene expression programmes driving cell-state transition while preventing activation of lineage-inappropriate genes.

BPCs are known to recruit PRC2 to deposit repressive histone marks^41,51,52^. We propose that these BPC-mediated epigenomic changes ‘lock in’ the paths to stomatal differentiation. Indeed, BPC1/2 are necessary and sufficient for H3K27me3 depositions within the *SPCH* locus (Fig. 4d,g,h). This is consistent with the previous findings that dysregulation of the H3K27me3 marks is associated with stomatal guard-cell reprogramming^35^. The stomatal-lineage chromatin accessibility sites undergo major reorganizations at or after the commitment stage (Fig. 1d,e). Thus, BPC1/2-MUTE-mediated epigenomic changes are probably followed by genome-wide chromatin remodelling. Our study suggests that timely upregulation of *MUTE* may be critical for ultimately driving the epigenomic landscape switch to stomatal differentiation. Previous molecular-genetic studies have suggested the role for the HD-ZIP IV family and other candidate transcription factors in promoting *MUTE* expression^64,65^. Yet, the direct mechanism of action remains unexplored. In any event, how differential actions of heterotypic TF groups, complexed with chromatin modifiers, guide the developmental progression at the atomic level is a future question to address.

## Methods

### Plant material and growth conditions

All plants used in this study are from *Arabidopsis thaliana* Columbia (Col-0) ecotype background. Seeds were stratified for 3 d at 4 °C and grown on half-strength Murashige and Skoog (½ MS) agar plates with 1% (w/v) sucrose. Plates were then placed under continuous light condition (24 h light, 80 µmol m^−2^ s^−1^) at a constant temperature of 21 °C. The following mutants and transgenic *Arabidopsis* lines have been described previously: the plasma membrane GFP marker Lti6b^66^; *bpc1‐1*, *bpc2, bpc1‐1 bpc2* series^40^; *iSPCH* and *iMUTE* estradiol-inducible lines^29^; *spch-3*^24^; *MUTEpro::nucYFP*^67^; *SPCHpro::SPCH-GFP*^23^

### Molecular cloning and generation of transgenic plants

Transgenic plants for INTACT carry two transgenes: (1) a constitutively expressed biotin ligase (*ACT2pro:BirA*) and (2) cell-type-specific-expressed Nuclear Tagging Fusion (NTF) protein containing a fusion of WPP nuclear envelope-targeting domain, a red fluorescent protein (RFP) for visualization and the biotin ligase recognition peptide (BLRP)^3^. To generate stomatal cell-state-specific NTF expression constructs, we implemented two modifications to a published vector^3^. First, the GFP cassette was replaced by RFP for enabling co-expression with existing GFP marker lines. Second, the R4L1 Gateway cloning system was adopted for rapid cloning^68^. For this purpose, NTF was cloned into modified L1L2 Gateway vector, pKUT612, and stomatal-lineage promoters (*AtML1, SPCH, MUTE, FAMA* and *GC1*) were cloned into pENTR 5′ TOPO system (Invitrogen). Three-way Gateway reaction was performed to generate *AtML1pro::NTF, SPCHpro::NTF, MUTEpro::NTF, FAMApro::NTF* and *GC1pro::NTF* constructs. These constructs were transformed into *Agrobacterium* GV3101(pMP90), and transgenic plants were generated by Agrobacterial floral dipping. At least 20 independent transgenic lines per construct were screened for monogenic segregation of transgene and specific expression patterns of RFP in protodermal and stomatal-lineage cells. The selected lines were crossed into established *ACT2pro::BirA* line^3^. The double homozygous lines were propagated to obtain a large quantity of seeds. For investigating the expression pattern of BPC1 and BPC2 during stomata development, *BPCpro1::BPC1-YFP* and *BPC2pro::BPC2-YFP* were generated by three-way Gateway reaction. Each promoter and gene body of *BPC* family was amplified and cloned into the pENTR 5′-TOPO and pENTR D-TOPO system (Invitrogen), and cloned into R4pGWB540^68^. The constructs were then transformed into the *Agrobacterium* strain GV3101, and transgenic plants were generated by Agrobacterial floral dipping. T1 plants were used in this study. For dual-luciferase transient assay, the double reporter vector and effector vector were generated. The double reporter includes an internal control REN (*Renilla* luciferase) driven by the 35S promoter of Cauliflower mosaic virus (CaMV) and firefly luciferase (LUC) driven by promoter of *SPCH* (−2,997 to −1) and promoter of *SPCH* (−2,997 to −1) with mutated GAGA motif (−2,331~−2,301, −172~−84, _53~−7). To construct the effector vector, the CDS sequence of each BPC family was amplified using Phusion Polymerase and cloned into pENTR D-TOPO. Using LR Gateway Recombination cloning methods, *35S*::*BPC1* and *35S*::*BPC2* were generated. For BiFC assays, split YFP constructs were generated by cloning the gene of interest into either the 35S::pSPYNE vector, which contains the N terminal of the EYFP protein or 35S::pSPYCE vector, which contains the C terminal of the EYFP protein using LR Gateway Recombination cloning methods^69^. The constructs were then transformed into the *Agrobacterium* strain GV3101, and co-infiltrated along with the reporter plasmid into *N. benthamiana* leaves. *SPCHpro::SPCH* and *SPCHmutGAGApro::SPCH* were also generated by three-way Gateway reaction. The promoter of *SPCH* (−2,997 to −1), promoter of *SPCH* (−2,997 to −1) with mutated GAGA motif (−2,331~−2,301, −172~−84, _53~−7) and the gene body of *SPCH* were cloned into the pENTR 5′-TOPO and pENTR D-TOPO system (Invitrogen), and then cloned into R4pGWB504^68^. For detailed information on plasmids generated in this study, see Supplementary Table 7. For a complete list of primer sequences used for cloning in this study, see Supplementary Table 8.

### Nuclei purification

Total nuclei were extracted from 3-day-old whole seedlings^70^. Briefly, 4–5 g of fresh tissue was harvested and finely ground in liquid nitrogen using a mortar and pestle. The resulting fine powder was transferred into 40 ml NPB (nuclear purification buffer containing 20 mM MOPS, 40 mM NaCl, 90 mM KCl, 2 mM EDTA pH 8, 0.5 mM EGTA, 0.2 mM Spermine, 0.5 mM Spermidine and protease inhibitor ×1) (Sigma 11873580001). The homogenized sample was filtered through a 120 µm mesh, then through a 70 µm mesh and lastly through a 40 µm mesh (Component Supply, U-CMN-120, 70, 40), and spun at 2,000 *g* for 10 min at 4 °C. The pellet was gently resuspended in 1 ml NPB, followed by incubation with streptavidin-coated Dynabeads (Invitrogen, 11205D, 00600299) for 30 min at 4 °C. The bead-nuclei mixture was gently washed in NPBt (NPB supplemented with 0.1% Triton X-100). After washing 3–4 times with NPBt, the bead-nuclei mixture was resuspended back in NPB. All steps were performed at 4 °C on ice. A fraction of the bead-nuclei mixture was subjected to DAPI staining (Sigma-Aldrich, D9542) and subsequently counted under a fluorescent microscope (Leica M165FC, Leica) to estimate the recovery of cell-state-specific nuclei as well as confirm the presence of intact nuclei. For the initial quality control (QC) of the INTACT method, qRT-PCR was performed to assess the fold change of *RFP* transcripts before and after Streptavidin-based affinity purification.

### ATAC-seq

All nuclei for ATAC-seq were freshly purified and never frozen. Transposition and library construction were performed as previously described^70^. In brief, 30,000 purified nuclei were used in each 50 µl transposition reaction for 30 min at 37 °C, with agitation using Nextera reagents (Illumina; FC-121-1030, 20191675). Transposed DNA fragments were purified with MinElute PCR purification kit (Qiagen, 28006) and then amplified using High Fidelity PCR Mix (NEB, M0541, 0061704) with custom bar-coded primer for 8–11 total PCR cycles. Amplified ATAC-seq libraries were purified using AMPure XP beads (Beckman Coulter, A63880, 16682800) and analysed using an Agilent 2100 Bioanalyzer to assess DNA quality as well as fragment size distribution before sequencing. INTACT ATAC-seq libraries were prepared using Nextera standard workflow and paired-end read (2 × 35 bp) in length on an Illumina Nextseq 400 system.

### ChIP

ChIP experiments were performed as previously described^47^, with minor modifications. The following antibodies that had previously been used for immunoprecipitation in *Arabidopsis* were used: anti-H3K27me3 (07-449, Millipore, 3669239) and anti-H3 (Abcam, ab17910), GR3398313-1 for *bpc1 bpc2*, *iBPC1* and *iBPC2* seedlings. For quantification of enrichment of H3K27me3 at *SPCH* loci, qPCR was performed and the enrichment of H3K27me3 was normalized to histone H3 to control for nucleosome density.

### ATAC-seq QC metrics

Enrichment at transcription start sites was computed as previously described^71^. Briefly, Tn5 cutcounts were summed at 1,000 bases up and downstream of all transcription start sites using the bedtools coverage function, and TSS enrichment was computed by taking, at each position, the ratio of cutcounts relative to the average cutcount in the flanking regions (100 bp average on either side). For all samples, TSS enrichment values showed a peak at the centre relative to the flanks. Cutcount values were normalized with DESeq2^72^ and used to compute pairwise sample correlations. Irreproducible discovery rate (IDR) was computed using the ‘idr’ package in R^73^.

### Peak calling and analysis across the stomatal development trajectory

Samples were sequenced on the Illumina NextSeq platform. Raw reads were mapped to the *Arabidopsis* genome (TAIR10 build) using bowtie2 with parameters defined using the --sensitive flag. Reads mapping to centromeric regions were removed. Reads were converted to bed format using bedtools and cut positions of all fragments <2,000 bp were determined from the endpoints of each fragment. Transposition events were aggregated for each sample using bedtools genomecov. Peaks were called using MACS2 with these parameters (macs2 callpeak -t your.bam -n yourbasename -f BAM --nomodel --shift −20 --extsize 150 --call-summits -g 1.3e08 -q 0.01 --bdg --outdir yourdir). We generated a union list of all THS peaks identified among stomatal-lineage samples combined with a previously published list of open chromatin regions from several *Arabidopsis* samples^10,11^. We were primarily interested in quantitative differences in accessibility in putative regulatory sites in the genome, hence we first merged the coordinates of the peak calls in the INTACT samples with previously identified regulatory sites^10^ using the bedtools merge function. This approach ensured that all samples were analysed using the same feature set and is akin to approaches used in single-cell ATAC-seq^74^ where regulatory sites (or peaks) are determined from combined data, but the counts, or quantitative measure of accessibility, are assessed per cell. The bedops function ‘bedmap’ (default parameters) was used to count the number of transposition events within each peak in each sample. Cutcounts per peak from each sample were combined and, to account for differences in total reads per sample, were normalized using the estimateSizeFactors() function in DESeq2 (R). We then used the Impulse software implementation within the DEseq2 framework^75^ to identify peaks whose cutcounts changed as a function of stomatal-lineage development (ordered: Proto → MMC → M → GMC → GC).

### Motif enrichment and clustering analysis

Using position weight matrices from a genome-wide in vitro binding study by DNA affinity purification sequencing (DAP-seq), we used FIMO to search for significant (adjusted *P* < 1 × 10^−5^) matches for each motif in accessible sites^36,37^. The output of this motif scan was used to generate a matrix that counts instances of each motif within each accessible site. Of 65,351 accessible sites, 58,478 (89.5%) had a significant motif match by FIMO. A high-dimensional matrix was generated that contains, for each THS peak, the number of counts for each motif identified in that region. The motif × peak count matrix was normalized according to the total size (in bp) of each peak, using size factors in the cell count data set object. The matrix was passed into Monocle3^14^, where we used UMAP dimensionality reduction (hyperparameters: min_dist = 0.01, n_neighbors = 25L), and the Leiden algorithm for identifying clusters of peaks with similar motif content. We examined the differentially accessible sites alone (Fig. 2e), and used the top_markers() function in Monocle3 to identify motifs with significant enrichment (adjusted *P* < 0.05) in MMC + M accessible sites vs GMC + GC accessible sites^14^. Differentially abundant motifs were analysed in peak clusters using the fit_models() function, which is a regression approach. We also grouped motif counts from THS peaks that showed differential accessibility in the ELC and compared those to motif counts in the PCCa; individual motifs were ranked according to their log_2_-fold enrichment in their respective cell-type-specific groups relative to the abundance of those motifs in ‘all’ THS peaks. Motif analyses of SPCH ChIP-seq and MUTE ChIP-seq were carried out by extracting the ±100 bp sequences from the peak centre and enriched motifs were identified using HOMER. Most statistically significant motifs were used for further analysis.

### UMAP analysis of total motif content in THSs

Output tables from the FIMO^36^ scan of DAP-seq motifs across stomatal THSs were used to generate a count matrix of all significant (*P* < 1 × 10^−5^) motif instances found across all THSs.To prevent artificial inflation of counts for motifs containing repetitive sequences, motifs only received a single count if they occurred more than once in a single THS. The count matrix was processed using Monocle3, where a size factor adjustment on motif counts for each THS was computed according to its length to minimize effects of THS size in subsequent calculations. The matrix was pre-processed using 10 PCA dimensions using the preprocess_cds() function, and subsequently fed into UMAP (hyperparameters: min_dist = 0.2, n_neighbors = 25) using the reduce_dimensions() function. Clusters of THS were identified using the cluster_cells() function with resolution = 1 × 10^−5^.

### Induced overexpression and quantitative RT-PCR

Homozygous transgenic lines for *iBPC1* and *iBPC2* were held at 4 °C for 4 d and then transferred to ½ MS media containing 10 μM Estradiol (Sigma, E8875) or DMSO (mock). Total RNA was isolated using (Millipore Sigma STRN50-1KT) and a DNase I digestion (Millipore Sigma DNASE70-1SET) was performed on column during the extraction according to manufacturer instructions. RNA (1 μg) was converted to complementary DNA using the iScript cDNA synthesis kit (Bio-Rad) following the manufacturer’s instructions. First strand cDNA was diluted 5-fold in double distilled water and used as a template for real time qPCR. qRT-PCR was performed as described previously^67^. For each experiment, three technical replicates were performed. Relative expression was calculated in relation to *ACTIN2* expression. See Supplementary Table 8 for details on the primer sequence used for qRT-PCR.

### Dual-luciferase transactivation assay

*N. benthamiana* was used for dual-luciferase assays in this study, as described previously^64^. A 35S- minimal promoter (−50 to −2) was fused with a firefly luciferase gene for basal gene expression. Promoter regions from *SPCH* (−2,998 to −1) and their mutated GAGA-motif version, were fused with *LUC* to generate reporter constructs. *CaMV35Spro::Ren* (*Renilla* luciferase) was used as a control. *Agrobacterium* carrying both effector and reporter constructs were infiltrated into 4–5-week-old *N. benthamiana*. At 5 d after infiltration, firefly and *Renilla* luciferase activities were assayed sequentially from a single sample using the Dual-Glo Luciferase Assay System kit (Promega, E2920). The assay was performed using a GloMax 96 microplate luminometer (Promega). See Supplementary Table 7 for detailed information on plasmids in this study.

### BiFC

BiFC assays were performed following a previously described method. Full coding sequences of *BPC1*, *BPC2*, *MUTE* and *SCRM* were inserted into a 35S::pSPYNE or 35S::pSPYCE Gateway recombination vector. The constructs were then transformed into the *Agrobacterium tumefaciens* strain GV3101 (pMP90). Cultured *Agrobacterium* in LB media were spun down and resuspended in infiltration buffer (10 mM MgCl2, 10 mM MES (pH 5.6) and 150 μM acetosyringone). Bacterial culture densities were adjusted to a final optical density at 600 nm (OD_600_) of 1.0, and the cell suspension was incubated at room temperature for 4 h before infiltration. Equal volumes of cultures carrying the corresponding complementary pair of BiFC constructs (YFPn and YFPc), along with a silencing suppressor plasmid-p19, were then co-infiltrated into 5-week-old *N. benthamiana* leaves. Leaf discs were collected 3 d after agro-infiltration. Capture of fluorescence images was performed using the sp8 Stella microscope (Leica). Images were captured from different regions of each inoculated leaf, and from at least two leaves per experiment. During post-acquisition processing, the images from each experiment were treated identically.

### Confocal microscopy

All confocal microscopy images were taken using a Zeiss LSM700, Leica SP5-WLL or Leica Stellaris 8 Falcon confocal microscope. Cell peripheries were visualized by staining with propidium iodide (Molecular Probes) with excitation at 619 nm and emission at 642 nm. Excitation at 488 nm and emission at 500–515 nm were used for visualizing the GFP signal, and excitation at 488 nm and emission at 490–546 nm were used for the YFP signal. For the Leica SP5-WLL, a HyD detector was used. The confocal images were false coloured, and their brightness/contrast were uniformly adjusted using Photoshop CS6 (Adobe).

### Quantitative analysis of stomata and stomatal-lineage cells

Stomatal density (number of stomata per mm^2^), SI and stomatal cluster distribution were quantified as described previously^76^. Quantitative analysis of stomatal cells was also performed as described previously^77^. Abaxial cotyledon epidermis from 8-day-old *Arabidopsis* seedlings was used for quantitative analysis of stomatal-lineage cells. All cell outlines in the image needed to be visible without damage. After adjusting brightness and contrast, cell outlines were filled manually. The different cell types were counted using ImageJ and processed with a script 36 that automatically quantify their surface areas. All images were taken using the Zeiss LSM700 confocal microscope.

### Yeast one-hybrid screen

Tandemly repeated DNA of THS motifs specific to SPCH/MUTE THSs (THS23375, 3X THS64787 and 5X THS61260) were commercially synthesized and sequence confirmed. The inserts were cloned into pHISi^45^. Subsequently, a high-throughput yeast one-hybrid screen was performed, employing 96-well plates and a robotic instrument; the library consisted only of *Arabidopsis* transcription factor genes, based on a previously described method^45^ as briefly described below. The promoter-reporter constructs (Pro::*HIS3*/pHISi) were individually integrated into the *URA3* locus of the YM4271 yeast strain (Clontech/Takara Bio) first to prepare the reporter yeast. Then their background activity was measured by spotting vector-transformed reporter yeast onto His-lacking media containing various concentrations of HIS3 inhibitor, 3-amino-1,2,4-triazole (3-AT, Sigma-Aldrich/Merck). The screens were performed in two steps using an automated robot (Freedom Evo 100, Tecan) against a prey library consisting of individually cloned, coding-region-only, sequence-verified clones for 1,736 genes for *Arabidopsis* transcription factors in pDEST-GADT7 vector^78^. The first assay was performed against the array of pools consisting of up to 6 prey clones, and the second assay was against single clones of the positive pools from the first assay. The extent of positiveness was classified manually into degrees of 1, 2 and 3 by a technical expert. After the secondary assay identified BPC proteins, the reporter yeast strains were re-transformed with prey BPC plasmids to confirm the interactions.

### Recombinant protein expression and purification

*A. thaliana* SCRM (ICE1) (1-494) was cloned into pGEX-4T-1 vector with an N-terminal GST tag and a thrombin cleavage sequence and MUTE (1-202), BPC1 (1-283) and BPC2 (1-279) were cloned to pET28a vector with N-terminal His tag. For protein expression, the constructs were transformed into *E. coli* strain BL21. For each transformant, a single clone was selected and incubated in 5 ml LB liquid medium. The overnight-incubated *E. coli* suspensions were transferred to 1 l LB medium and incubated at 37 °C for around 2 h until the OD_600_ reached 0.4–0.6. The isopropyl *β*-d-1-thiogalactopyranoside (0.25 μM of final concentration) was added to the cultures and the strains were incubated at 25 °C for a further 16 h.

GST-fused proteins were purified using glutathione agarose resin, and His tag proteins were purified using Ni-NTA agarose resin. The soluble portion of the cell lysate was loaded onto a GST-Sepharose column. Non-specifically bound proteins were removed by washing the column with 20 mM Tris pH 8.0 and 200 mM NaCl. The bound GST-fused protein was eluted with 10 mM glutathione, 20 mM Tris and 200 mM NaCl (pH 8.0). The GST-fused proteins were exchanged with phosphate-buffered saline buffer, and then the solution was treated with 50 μg of thrombin for 10–12 h at 16 ^ο^C. The GST portion of the protein was cleaved during thrombin digestion, and then the whole solution was reloaded onto the glutathione *S*-transferase column to obtain pure protein. The purified proteins were further purified by gel filtration on a Superdex-200 (GE) column using fast protein liquid chromatography and phosphate buffer (pH 7.2) as the eluent. BPC1, BPC2 and MUTE N-terminal His tagged proteins were purified using an Ni-NTA column, followed by gel filtration on a Superdex-200 (GE) column using fast protein liquid chromatography and phosphate buffer (pH 7.2) as the eluent.

### Biotinylation of DNA oligomers

DNA oligomers were biotinylated at the 3′ end with a DNA 3′-end biotinylation kit (Pierce, Thermo Fisher). In a typical 100 ml reaction, about 50 nM of purified DNA was mixed with Biotin-11-UTP and terminal deoxynucleotidyl transferase (TdT) in the reaction buffer. After incubation of the mixture at 37 °C for 30 min, the reaction mixture was washed with an excess amount of chloroform: isoamylalcohol (24:1) mixture to remove TdT, and then purified DNA was collected from the aqueous phase. To prepare the dsDNA, 3′-biotin-E-box/GAGA oligos, biotinylated samples were annealed to its complementary strand in 1x binding buffer (PBS buffer pH 7.3) at 90 °C for 5 min and cooled to room temperature.

### BLI

The binding affinities of the BPC1 and BPC2 proteins with SCRM, SPCH, MUTE, SCRM-MUTE heterodimer and SCRM-SPCH heterodimer were measured using the Octet Red96 system (ForteBio, Pall Life Sciences) following the manufacturer’s protocols. The optical probes coated with Ni-NTA were first loaded with 500 nM His tagged BPC1 and BPC2 before kinetic binding analyses. The experiment was performed in 96-well plates maintained at 30 °C. Each well was loaded with 200 μl reaction volume for the experiment. The binding buffer used in these experiments contained 1× PBS supplemented with 0.02% Tween 20. The concentrations of the SCRM, SPCH, MUTE, SCRM-MUTE heterodimer and SCRM-SPCH as the analyte in the binding buffer were 200 nM, 100 nM, 50 nM, 25 nM, 12.5 nM, 6.25 nM and 3.12 nM. There was no binding of the analytes to the unloaded probes as shown by the control wells. Binding kinetics to all seven concentrations of the analytes were measured simultaneously using default parameters on the instrument.

The binding affinities of the SCRM-SPCH, SCRM-MUTE, BPC1 and BPC2 proteins with biotinylated GAGA and E-box repeats of nucleotides were measured using the Octet Red96 system. The optical probes coated with streptavidin were first loaded with 1 nM GAGA motif/E-box motif before kinetic binding analyses. The experiment was performed in 96-well plates maintained at 30 °C. Each well was loaded with 200 μl reaction volume for the experiment. The binding buffer used in these experiments contained 1× PBS supplemented with 0.02% Tween 20. The concentrations of the BPC1, BPC2, SCRM-SPCH heterodimer and SCRM-MUTE heterodimer as the analyte in the binding buffer were 500 nM, 250 nM, 125 nM, 62.5 nM, 31.25 nM, 15.62 nM and 7.81 nM. Binding kinetics to all seven concentrations of the analytes were measured simultaneously using default parameters on the instrument. The data were analysed using the Octet data analysis software. The association and dissociation curves were fit with the 1:1 homogeneous ligand model. The kobs (observed rate constant) values were used to calculate the dissociation constant (*K*_d_), with steady-state analysis of direct binding.

### Reporting summary

Further information on research design is available in the Nature Portfolio Reporting Summary linked to this article.

## Supplementary information

Reporting Summary

Supplementary Tables Supplementary Table 1. INTACT ATAC-seq library seq information. Table 2. Stomata union THS peak coordinates. Table 3. Dynamic THSs peak coordinates. Table 4. SPCH ChIP-seq peak and target gene with bHLH and BBR/BPC motif. Table 5. MUTE ChIP-seq peak and target gene with bHLH and BBR/BPC motif. Table 6. ELC and PCC THSs list bound by either SPCH or MUTE. Table 7. Plasmid construct generated or used in this study. Table 8. Primers and DNA fragment sequence used for this study. Table 9. Exact *P* values for statistical analysis.

## Data Availability

The INTACT ATAC-seq NGS data are available at NCBI GEO (accession number: GSE190753).

## Code availability

Analysis pipelines and data files can be found in Github (https://github.com/ToriiLab/Stomatal-Lineage-ATAC-seq).
