# Supplementary material for: Dynamic chromatin accessibility deploys heterotypic cis/trans-acting factors driving stomatal cell-fate commitment
Source: Nat Plants. 2022 Dec 15;8(12):1453–66. doi: 10.1038/s41477-022-01304-w (PMC9788986; doi:10.1038/s41477-022-01304-w)
Supplement: Supplementary file 1 — Reporting Summary [file 41477_2022_1304_MOESM1_ESM.pdf]

nature portfolio

Corresponding author(s): TORII, Keiko U.

Last updated by author(s): Oct 24, 2022

Reporting Summary

Nature Portfolio wishes to improve the reproducibility of the work that we publish. This form provides structure for consistency and transparency in reporting. For further information on Nature Portfolio policies, see our Editorial Policies and the Editorial Policy Checklist.

Statistics

For all statistical analyses, confirm that the following items are present in the figure legend, table legend, main text, or Methods section.

n/a	Confirmed	
☐	☒	The exact sample size (*n*) for each experimental group/condition, given as a discrete number and unit of measurement
☐	☒	A statement on whether measurements were taken from distinct samples or whether the same sample was measured repeatedly
☐	☒	The statistical test(s) used AND whether they are one- or two-sided<br>*Only common tests should be described solely by name; describe more complex techniques in the Methods section.*
☐	☒	A description of all covariates tested
☐	☒	A description of any assumptions or corrections, such as tests of normality and adjustment for multiple comparisons
☐	☒	A full description of the statistical parameters including central tendency (e.g. means) or other basic estimates (e.g. regression coefficient) AND variation (e.g. standard deviation) or associated estimates of uncertainty (e.g. confidence intervals)
☐	☒	For null hypothesis testing, the test statistic (e.g. F, t, r) with confidence intervals, effect sizes, degrees of freedom and P value noted<br>*Give P values as exact values whenever suitable.*
☒	☐	For Bayesian analysis, information on the choice of priors and Markov chain Monte Carlo settings
☒	☐	For hierarchical and complex designs, identification of the appropriate level for tests and full reporting of outcomes
☐	☒	Estimates of effect sizes (e.g. Cohen's *d*, Pearson's *r*), indicating how they were calculated

Our web collection on statistics for biologists contains articles on many of the points above.

Software and code

Policy information about availability of computer code

Data collection	No software was used in data collection; ATAC-seq datasets were generated in this study.
Data analysis	Processing ATAC-seq data: bcl2fastq (v2.18), bowtie2 (v2.4.1), bedtools (2.25.0). MACS2 (2.1.1.), Dynamic peaks: ImpulseDE2 (v1.11.0). Motif analysis was conducted using modified Monocle3 (v1.0.1) pipeline in R(v4.0.2); code and data files can be found at https://github.com/ToriiLab/Stomatal-Lineage-ATAC-seq.

For manuscripts utilizing custom algorithms or software that are central to the research but not yet described in published literature, software must be made available to editors and reviewers. We strongly encourage code deposition in a community repository (e.g. GitHub). See the Nature Portfolio guidelines for submitting code & software for further information.

Data

Policy information about availability of data

All manuscripts must include a data availability statement. This statement should provide the following information, where applicable:

- Accession codes, unique identifiers, or web links for publicly available datasets
- A description of any restrictions on data availability
- For clinical datasets or third party data, please ensure that the statement adheres to our policy

The authors declare that the data supporting the findings of this study are available within the paper and its supplementary information files. All the sequencing data generated in this work has been deposited to NCBI GEO (accession number: GSE190753). Analysis pipelines and data files can be found at Github (https://github.com/ToriiLab/Stomatal-Lineage-ATAC-seq).

Field-specific reporting

Please select the one below that is the best fit for your research. If you are not sure, read the appropriate sections before making your selection.

☒ Life sciences ☐ Behavioural & social sciences ☐ Ecological, evolutionary & environmental sciences

For a reference copy of the document with all sections, see nature.com/documents/nr-reporting-summary-flat.pdf

Life sciences study design

All studies must disclose on these points even when the disclosure is negative.

Sample size	Sample size was determined based on similar studies in this field. For stomatal index analysis, the sample size was at least 6<br>For ATACseq, no explicit sample-size calculation was performed for number of seedlings, but individual-INTACT-ATACseq protocols were optimized to sample enough seedling to capture enough lineage specific nuclei between 20,000-60,000
Data exclusions	No data were excluded
Replication	All experiments were performed using at least two biological replicated with at least three technical replicates with highly reproducible results.<br>INTACT ATAC-seq on tissues of various genotypes were performed at least 3-4 replicates.
Randomization	Plants for all phenotypic characterization were randomly chosen among each genotype population. Positions of plants in pots and petri dishes were exchanged every day to minimize the effect of environmental variability in the growth room.
Blinding	No blinding was performed in this study. This is because blinding requires mixing seeds of different genotypes (mutants and transgenic lines), which could lead to a risk of mislabeling.

Reporting for specific materials, systems and methods

We require information from authors about some types of materials, experimental systems and methods used in many studies. Here, indicate whether each material, system or method listed is relevant to your study. If you are not sure if a list item applies to your research, read the appropriate section before selecting a response.

Materials & experimental systems

n/a	Involved in the study	
☐	☒	Antibodies
☒	☐	Eukaryotic cell lines
☒	☐	Palaeontology and archaeology
☒	☐	Animals and other organisms
☒	☐	Human research participants
☒	☐	Clinical data
☒	☐	Dual use research of concern

Methods

n/a	Involved in the study	
☒	☐	ChIP-seq
☒	☐	Flow cytometry
☒	☐	MRI-based neuroimaging

Antibodies

Antibodies used	H3K27me3 (07-449, Millipore, Lot 3669239) and anti-H3 (Abcam ab17910, Lot GR3398313-1 are used for ChIP. Both of them are used 1:1000 dilution
Validation	anti-H3K27me3 and anti-H3 antibodies were commercially available and the data sheets were provided by companies. These antibodies are recommended for IP (Immunoprecipitation) assay including ChIP. Both antibodies were described in a previous publication (Kim et al.,2021 and Xiao et al.,2017)<br>https://www.abcam.com/histone-h3-antibody-nuclear-marker-and-chip-grade-ab1791.html<br>https://www.emdmillipore.com/US/en/product/Anti-trimethyl-Histone-H3-Lys27-Antibody,MM_NF-07-449

March 2021
